# Supplementary material for: Cadherin-11-Interleukin-6 Signaling between Cardiac Fibroblast and Cardiomyocyte Promotes Ventricular Remodeling in a Mouse Pressure Overload-Induced Heart Failure Model
Source: Int J Mol Sci. 2023 Mar 31;24(7):6549. doi: 10.3390/ijms24076549 (PMC10094910; doi:10.3390/ijms24076549)
Supplement: Supplementary file 1 [file ijms-24-06549-s001.zip › Table S1.pdf]

Table S1. Sequences and downstream primers for genes analyzed by qRT-PCR (h- human, m-mouse).

Gene	Forward primer	Reverse primer
Cad-11(h)	5′-ATGGCTCAGGCGGTGGACAG-3′	5′-GGCACGTTGGCATGATAGGTCTC-3′
GAPDH(h)	5′-CCAGAACATCATCCCTGCCT-3′	5′-CCTGCTTCACCACCTTCTTG-3′
Cad-11 (m)	5′-ATGGCTCAGGCGGTGGACAG-3′	5′-CATTGGACCTCTCAGGCACATTGG-3′
ANP(m)	5′-CGAGGTGCCTCCCTGGACTG-3′	5′-GCGAGCAGAGCCCTCAGTTTG-3′
BNP(m)	5′-CCTTCGGTCTCAAGGCAGCAC-3′	5′-CAGCCCAAACGACTGACGGATC-3′
β-MHC(m)	5′-GGAGACACGCAGCCGCAATG-3′	5′-GCCTCAGCAGCCATACGGTTG-3′
GAPDH (m)	5′-AGGTCGGTGTGAACGGATTTG-3′	5′-TGTAGACCATGTAGTTGAGGTCA-3′
Cav1.2(m)	5′-CCGCAGCGTAAGGATGAGTGAAG-3′	5′-GGCAGGTCAGTTGTCGGTGTTC-3′
Kv4.2(m)	5′-GACATGGCAAGACACCCTGG-3′	5′-TTGTTGGGTCTCTGGGTGGT-3′
Kv4.3(m)	5′-CAGCGGTGTCCTGGTCATTGC-3′	5′-GCCCTGCGTTTATCTGCTCTCTG-3′
KCHIP2(m)	5′-AGCGGAATTGTCAACGAGGAGAAC-3′	5′-AGCCATCATGGTTGGTGTCAAAGG-3′
Kv1.5(m)	5′-TGGCTCTGGTGTCCTTTCCTCTG-3′	5′-GGGCAAGCAAAGAAACGCACAAG-3′
Kv2.1(m)	5′-GGAAATGGACCAAGAGGGCG-3′	5′-CAAGTGCTGCGGACTAGACG-3′
MIF (m)	5′-GCATCGGCAAGATCGGTGGTG-3′	5′-ACGTTGGCAGCGTTCATGTCG-3′
IL-6 (m)	5′-ACTTCCATCCAGTTGCCTTCTTGG-3′	5′-TTAAGCCTCCGACTTGTGAAGTGG-3′
TNF-α(m)	5′-GCCTCTTCTCATTCCTGCTTGTGG-3′	5′-GTGGTTTGTGAGTGTGAGGGTCTG -3′
